# The effect of nanodiamonds on candida albicans adhesion and surface characteristics of PMMA denture base material - an *in vitro* study

**DOI:** 10.1590/1678-7757-2018-0779

**Published:** 2019-11-04

**Authors:** Shaimaa M. Fouda, Mohammed M. Gad, Passent Ellakany, Ahmad M. Al-Thobity, Fahad A. Al-Harbi, Jorma I. Virtanen, Aune Raustia

**Affiliations:** 1 Department of Substitutive Dental Sciences, College of Dentistry, Imam Abdulrahman Bin Faisal University, Dammam, Saudi Arabia; 2 Department of Community Dentistry, Faculty of Medicine, University of Turku, Turku, Finland; 3 Research Unit of Oral Health Sciences, Prosthetic Dentistry and Stomatognathic Physiology, Faculty of Medicine, University of Oulu, Oulu, Finland; 4 Medical Research Center Oulu, Oulu University Hospital and University of Oulu, Oulu, Finland

**Keywords:** *Candida albicans*, Denture stomatitis, PMMA, Nanodiamonds, Denture base

## Abstract

*Candida albicans* is the main causative pathogen of denture stomatitis, which affects many complete denture patients. Objective: To evaluate the effect of different concentrations of nanodiamonds (NDs) added to polymethyl methacrylate (PMMA) denture base material on *Candida albicans* adhesion as well as on surface roughness and contact angle. Methodology: Acrylic resin specimens sized 10×10×3 mm^3^ were prepared and divided into four groups (n=30) according to ND concentration (0%, 0.5%, 1%, 1.5% by wt). Surface roughness was measured with a profilometer, and the contact angle with a goniometer. The effect of NDs on *Candida albicans* adhesion was evaluated using two methods: 1) slide count and 2) direct culture test. Analysis of variance (ANOVA) and Tukey's post hoc test were used in the statistical analyses. Results: Addition of NDs decreased the *Candida albicans* count significantly more than in the control group (p<0.05), with a lowest of 1% NDs. Addition of NDs also significantly decreased the surface roughness (p<0.05), but the contact angle remained the same. Incorporation of NDs into the PMMA denture base material effectively reduced *Candida albicans* adhesion and decreased surface roughness. Conclusion: PMMA/NDs composites could be valuable in the prevention of denture stomatitis, which is considered one of the most common clinical problems among removable denture wearers.

## Introduction

Edentulism increases in old age,^1^ and in such cases, a conventional complete denture is commonly the treatment of choice. Denture bases are constructed from metal and/or acrylic resin. Acrylic resin, however, is more frequently used due to its ease of construction and repair, aesthetics and low cost, despite the material's drawbacks of high surface roughness and low strength.^2^ A study shows that denture stomatitis (DS) affects more than 70% of patients wearing complete dentures.^3^ Many factors, such as poor oral hygiene, poor fitting dentures, roughness, porous denture surfaces and systemic diseases, are associated with DS, of which *Candida albicans* is considered the main causative pathogen.^3^ Studies found that hydrophobicity and surface roughness of denture bases affect the primary attachment and colonization of *Candida albicans*.^3^^,^^4^ Therefore, to reduce *Candida albicans* adherence, the denture base material should be hydrophilic, smooth and less porous.^3^^,^^4^

Examples of ways to reduce the incidence of DS are mechanical cleansing, chemical disinfection, antifungal agents, surface modifications, and/or incorporating antimicrobial agents in the denture base material.^5^^–^^8^ Conventional methods used to clean dentures are usually effective at eliminating plaque accumulation.^8^ Their application, however, may be challenging for older patients, particularly those with physical disabilities or in need of nursing care. Oral antifungal agents are effective in the treatment of DS, but have toxic side effects and may lead to the development of resistant strains. In addition, DS recurrence commonly occurs with their use.^9^

Antimicrobial effect of chemical disinfectants is related to their proper use according to the preparation guidelines and immersion time.^10^ Morever, studies found that their use adversely affect the physical properties of denture cleansers, leading to increased surface roughness, color changes and reduced flexural strength.^10^^,^^11^ Therefore, many studies have investigated the effect of adding antimicrobial/antifungal agents to denture base resin in an attempt to reduce microbial and/or fungal adhesion and thereby prevent DS.^12^^–^^14^

Surface roughness (Ra) and hydrophobicity are important properties of the denture base material that influence plaque, microbial adhesion and, subsequently, DS.^15^^,^^16^ A rough denture surface provides more area for microbial adhesion. In addition, it protects entrapped microorganisms from shearing forces during denture cleaning, making their removal difficult even when using antimicrobial agents.^17^^,^^18^ High hydrophobicity of denture surfaces increases *Candida albicans* adhesion due to the hydrophobic interaction between it and the denture base resin.^18^ To avoid increased microbial colonization, Ra should be inferior to 0.2 μm.^16^ Recently, the addition of nanoparticles to polymethyl methacrylate (PMMA) has attracted attention because they enhance the mechanical and physical properties of the resin as well as its antimicrobial resistance.^19^ Antimicrobial effect is related to the surface energy of nanoparticles and chemical reactivity.^20^ Several nanosized materials, such as silver, platinum, and titanium, have been added to PMMA, resulting in better resistance to bacterial and fungal colonization.^19^

Nanocarbon family has superior physical and chemical properties and antibacterial effect.^21^ Nanodiamonds (NDs) belong to the nanocarbon family and have advantages over other metal/metal-oxide nanoparticles for being more chemically stable, biocompatible, and not inducing cytotoxicity.^22^ They are used in different fields of Medicine and Dentistry, including guided tissue regeneration, polymer reinforcement, and antibacterial dental implant coatings.^22^^,^^23^

NDs has multiple reactive groups (NH_2_, OH) that improve their interfacial bond with PMMA, and are thus considered a compatible filler material.^24^^,^^25^ A number of studies confirmed the antibacterial activity of NDs.^26^^,^^27^ Wehling, et al.^26^ (2014) suggested that the bactericidal effect of NDs is related to the presence of oxygen-derived groups on its surface. Reaction between these surface groups and components of bacterial cells may be the cause of antibacterial activity of NDs.^27^ Al-Harbi, et al.^28^ (2019) reported improvement in the mechanical properties of ND-reinforced PMMA. Although authors have reported the antimicrobial effect of NDs, none have investigated their effect against *Candida albicans* adhesion. This study evaluated the effect of different concentrations of NDs incorporated into PMMA denture base material on *Candida albicans* adhesion, as well as on the surface and contact angle, with the purpose of preventing denture stomatitis. The first research hypothesis predicted that the surface roughness or contact angle of the PMMA denture base material would be unaffected by the different concentrations of NDs. The second research hypothesis predicted that when assessed with two different antifungal assays, the PMMA denture base resin containing different concentrations of NDs would show no antifungal effect against *Candida albicans*.

## Methodology

The study was conducted between February 2018 and July 2018. Specimens were fabricated at the Prosthodontics Laboratory and tested at the Research Laboratory, College of Dentistry, while microbiology assays were carried out at the Department of Microbiology, College of Medicine, and Scanning electron microscopy (SEM) at the Electron Microscopy Unit, Institute for Research and Medical Consultations, Imam Abdulrahman Bin Faisal University.

ND/PMMA composite preparation: The ND powder (Shanghai Richem International Co. Ltd) used in this analysis has an average particle size of 30 nm. Transmission electron microscopy (TEM) was used to assess the surface morphological features of the powder, which showed that the powder consisted of graphite sheets (56% wt) varying from a few to several tens of nanometers and NDs (44% wt) with an estimated particle size of around 30 nm. NDs were weighed with an electronic balance (S-234, Denver instrument) in concentrations of 0.5, 1, and 1.5% of the acrylic resin powder.^28^ The mix was stirred first with gentle hand pressure using a conventional mortar and pestle, then with an electric mixer for 30 min at 400 rpm to ensure equal dispersion of the filler in the resin powder.^28^

### 

#### Specimen fabrication

**Figure 1:**
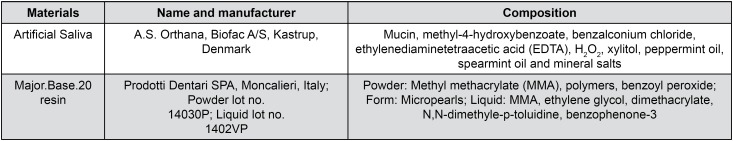
Composition of heat cured acrylic resin and artificial saliva

Heat-polymerized acrylic resin (Figure 1 Major base 20 resin; Prodotti Dentari SPA) was used to fabricate 120 specimens. The specimens were divided into four groups according to ND concentration, each including 30 specimens. Metal molds were used to fabricate wax specimens with 10×10×3 mm^3^ in size, then the wax specimens were invested in dental stone (Fujirock EP; GC) within flasks (61B Two Flask Compress; Handler Manufacturing). After the setting of the stone was complete, the flasks were placed into a wax elimination machine for five minutes to dissolve the wax. The resulting mold spaces and all the stone surfaces were coated with a separating medium (Isol Major; Major Prodotti Dentari Spa). A porcelain jar was used for mixing the polymer and monomer according to the manufacturer's guidelines. When the mix reached the dough stage, it was hand-kneaded, then packed and processed in a heat-curing unit (KaVo Elektrotechnisches Werk GmbH, Leutkirch, Germany) for two hours at 74°C, followed by one hour at 100°C. Before deflasking, the flasks were left to cool at room temperature. The specimens were finished and polished with a tungsten carbide bur (HM 79GX-040 HP; Meisinger) with a thin cross cut at 18 000 rpm, followed by a fine-grain cylindrical rubber top bur for the acrylic resin (HM251FX-040-HP; Meisinger).^28^ To standardize the polishing procedures, definitive polishing on a polishing cloth disc (TexMet C10in, 42-3210, Buehler GmbH) was carried out with a mechanical polisher (Metaserve 250 grinder-polisher, Buehler) at 100 rpm for five minutes in wet conditions.^28^ After ultrasonic cleaning, the specimens were incubated for one week at 37°C in distilled water, which was changed daily to reduce accumulation of residual monomers.^29^

#### Surface roughness test

A non-contact optical interferometric profilometer (Contour Gt-K1 optical profiler; Bruker Nano, Inc., Tucson, AZ) was used to measure the Ra of the specimens at a 0.01 mm resolution. The specimens (approximate area 0.43×0.58 mm) were scanned with a standard camera at 20× at five sites, and the average for each specimen was calculated. A software package (Vision64, Bruker Nano) was used to analyze the acquired images. Pit characteristics were determined, and the Ra value of each specimen was calculated.

#### Scanning electron microscopy (SEM)

Scanning electron microscope (SEM) (FEI, ISPECT S50) was used to examine the specimens’ surface characteristics (topography). To avoid the non-conductive property of the material, a gold coating was applied with a sputter coating machine (Quorum, Q150R ES, UK). Images were captured at different magnifications (500, 1000, 2000, 5000, and 10000×) to observe important characteristics of the surface changes with different ND concentrations.

#### Contact angle measurement

After surface roughness test, the surfaces of specimens were gently dried with air. Droplets of distilled water were applied on the surface of the specimens using an auto pipette and a goniometer to standardize the droplets volume (2 μL). An automated contact angle goniometer (DM-501; Kyowa Interface Science Co, Japan) was used to measure the contact angle. The angle of the tangent to the surface of the water droplet was measured and repeated four times on different areas of each specimen. Thereafter, the average was calculated. The images were analyzed with FAMAS software (Kyowa Interface Science Co, Japan).

#### Microbiology test

The specimens were sterilized with 70% alcohol, then cleaned ultrasonically with sterilized distilled water.^29^ The sterilized specimens were soaked in artificial saliva (Figure 1) containing 2 million cells of *Candida albicans* (ATCC 10231) at 37°C for 48 hours.^6^^,^^7^^,^^30^ To detach non-adherent cells, phosphate-buffered saline (PBS) was used to wash acrylic plates three times. The plates were then put into sterile tubes containing 1 ml of Sabouraud's dextrose broth (SDB – Acumedica Co., Manufacturers, Inc.) for 24 hours. After that, the plates were vibrated for 10 minutes with a vortex mixer. To obtain clustered pellets of *Candida albicans*, the tubes were then centrifuged for five minutes at 4500 rpm.

#### Evaluation

After being centrifuged, the acrylic resin specimens were extracted from the tubes, and the clustered pellets were collected from the tube. The *Candida albicans* attached to each specimen was counted by two methods:^6^^,^^7^^,^^30^

1. Slide count method (Neubauer): For microscopic evaluation, 2.5 µl of Trypan Blue 0.4% solution in phosphate (MP-Biomedicals) was added to 7.5 *µl* of each concentrated *Candida* pellet of the specimen positioned on a slide worktable (Neubauer Slide Counter; Chambers-Marienfeld). The Trypan Blue stain distinguishes the living *Candida albicans* from the dead by showing the living *Candida albicans* cells as transparent and surrounded by a blue borderline, while the dead cells were colored blue. A light microscope (at low power magnifications,10*×*) was used to count the number of *Candida albicans* cells. Each slide contained four main squares, each square was divided into 16 smaller squares. *Candida albicans* cells were counted in two main squares, then multiplied by two to achieve the total number of *Candida albicans* on each slide.^6^^,^^7^
2. Direct culture method [colony-forming unit (CFU)]: 10 µl of each isolated centrifuged pellet was spread onto a petri dish and incubated for 24 hours at 37°C.^30^ A marker pen counter (colony counter “SP Scienceware, Bel-Art Products”) was used to count the *Candida albicans* colonies. The number of colonies was calibrated for the dilution factor. When the number of colonies reached 5000 or more, it was considered overgrown.

### Statistical analysis

We used IBM SPSS Statistics 23 (IBM Corp., Armonk, NY) for all statistical analyses. Arithmetic means and standard deviations for categorized parameters were calculated. Analysis of Variance (ANOVA) was used to check overall significance, and pairwise significance was tested by Tukey's *post hoc* test. The level of significance was set at p< 0.05.

## Results

**Figure 2:**
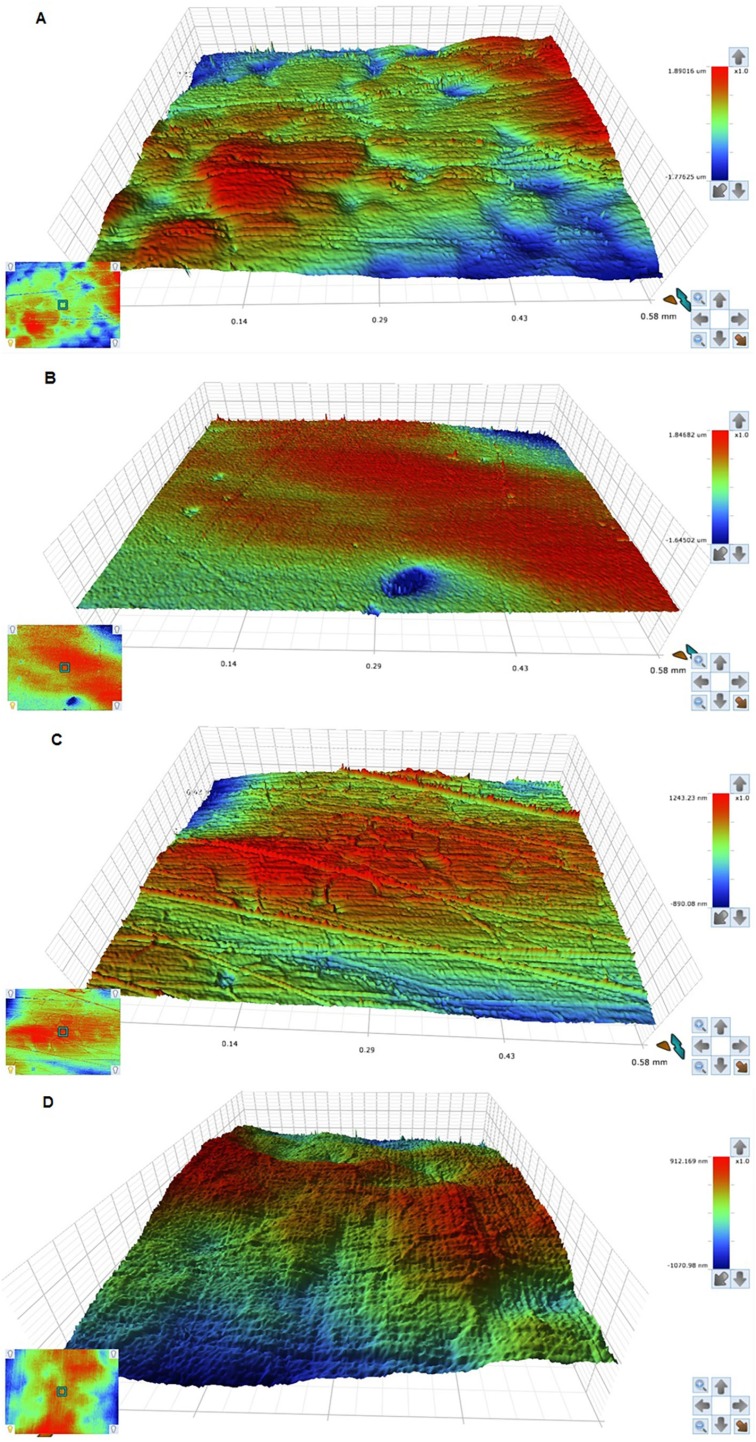
Representative surface images of: (A) Unmodified specimens; (B) 0.5% ND concentration; (C) 1% ND concentration; (D) 1.5% ND concentration.

**Figure 3:**
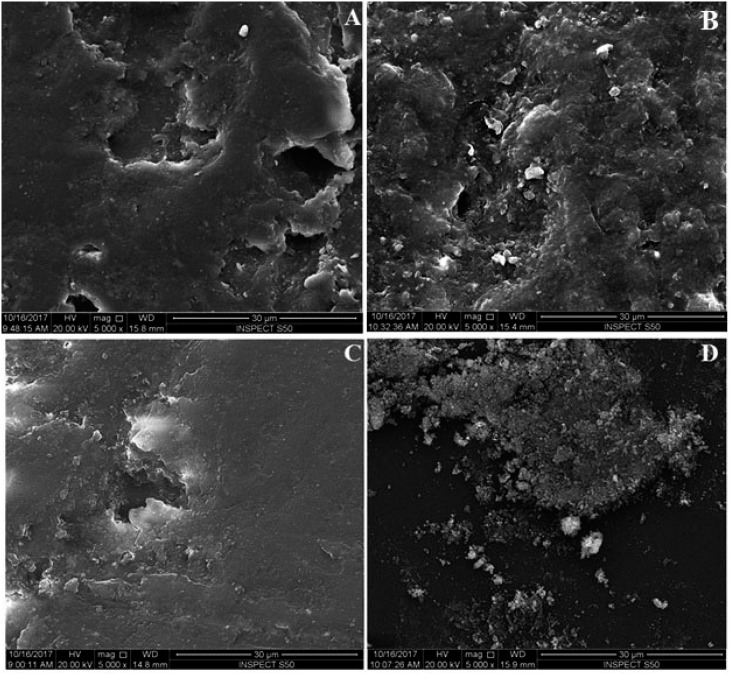
Representative SEM images of the tested specimens’ surfaces. (A) Unmodified specimens; (B) 0.5% ND concentration; (C) 1% ND concentration; (D) 1.5% ND concentration

In comparison to the control group, the addition of NDs significantly reduced the surface roughness (p<0.05) (Table 1). The highest Ra value was from the control group (0.129±0.011 µm), whereas the lowest value was with 0.5% NDs group (0.039±0.009 µm) with no significant difference between 0.5% NDs and 1% NDs groups (p*=0.396)*. Significant differences were found between the 0.5% NDs and 1% NDs groups and the 1.5% NDs group (p<0.05), which presented the highest Ra value of the NDs groups. Figure 2 shows the color parameter representing the average Ra values, which range from red at the parameter top to blue at the parameter bottom. Where rough surfaces have peaks, valleys, and areas in between, red reveal the peaks while blue reveals the valley depth, and the interdigitated colors in between exhibited areas between the peaks and valleys. Thus, the graduated color of this parameter displayed the whole surface roughness. Figure 3 shows SEM micrographs of the surface of the specimens at 5000× magnification with different concentrations of NDs. SEM analysis displayed high Ra in unreinforced heat-cured acrylic resin (Figure 3A) containing broad scattered pores with dimensions of 80 to 100 microns and depth variations. Figure 3B (0.5% NDs) shows a compact morphology with diminutive pores preoccupied by NDs measuring a few microns in diameter. Figures 3C (1% NDs) and 3D (1.5% NDs) show a smooth surface resulting from NDs filling the pores. Figure 3D shows loosely-attached clusters of NDs on the surface of specimens.

**Table 1:** Mean and standard deviations of surface roughness of studied group

Group	Control	0.5% ND	1%ND	1.5%ND
Mean±SD	0.129±0.011	0.039±0.009A	0.047±0.007A	0.102±0.017
	f-value (149.089)		p-value (0.000)	

F = ANOVA test, One-way ANOVA and Tukey's post hoc test (p<0.05) indicate differences among NDs concentrations as shown by superscripts letter; the same letter indicates no significant difference.

Table 2 shows the mean values of the contact angles in each group against distilled water. No significant difference was found in contact angles when comparing all tested groups (p=0.083) or the control group with the NDs-reinforced groups (p>0.05). The highest contact angle value was 84.8±1.62, recorded with the 1.5% NDs group, while the lowest contact angle value was 82.4±1.8, recorded with the 0.5% NDs group.

**Table 2:** Mean and standard deviations of contact angles in all tested groups

Group	Control	0.5% ND	1%ND	1.5%ND
Mean±SD	82.9±3.14^A^	82.4±1.8^A^	83±1.63^A^	84.8±1.62^A^
	f-value (11.025)		p-value (0.083)	

F = ANOVA test, One-way ANOVA and Tukey's post hoc test (p<0.05) indicate differences among NDs concentrations as shown by superscripts letter; the same letter indicates no significant difference.

Table 3 presents the means and standard deviations of all tested groups regarding the Neubauer and CFU tests. Referring to Neubauer, the *Candida albicans* count in the control group had a significant increase compared with the other NDs groups (p<0.05), the lowest *Candida albicans* count occurred in the 1% NDs group (276.2±34.47). In the NDs groups, the 0.5% NDs group presented a significant difference compared with the 1% and 1.5% NDs groups, while the 1% NDs and 1.5% NDs groups showed no significant difference between them.

**Table 3:** Mean and standard deviations of different studied groups regarding slide count (Neubauer) and Colony forming unit (CFU)

Group	Neubauer	CFU
Control	2035.9±121.03^a^	13212.0±821.2^A^
0.5 ND	1039.4±88.11^b^	7604.7±310.97^B^
1 ND	276.2±34.47^c^	2848.6±187.8^C^
1.5 ND	303.3±35.93^cd^	3602.0±339.25^CD^
F	21.36	104.2
P	0.01*	0.0001*

F = ANOVA test, One-way ANOVA and Tukey's post hoc test (p<0.05) indicate differences among NDs concentrations as shown by differences in superscripts letter vertically; the same letter indicates no significant difference. Lower case letters indicate slide count test while uppercase letters indicate cell culture count.

The number of living *Candida albicans* cells was obtained with a culture test. The CFU method showed a significant increase in the *Candida albicans* count in the control group compared to the NDs groups (p<0.05), with the lowest CFU count occurring in the 1% NDs group (2848.6±187.8). In the NDs groups, the 0.5% NDs group showed a significant difference compared with the 1% and 1.5% NDs groups, while the 1% and 1.5% NDs groups had no significant difference between them. The number of *Candida albicans* observed with the CFU test decreased significantly with the addition of NDs, especially in the 1% and 1.5% NDs groups. The Neubauer and CFU test methods had no statistically significant difference, which confirmed the effect of NDs against *Candida albicans*, as their effect was approximately the same regardless of concentration.

## Discussion

A denture base resin with antifungal properties would be useful to prevent DS and helpful for complete denture wearers who face difficulty in cleaning their dentures properly. The results showed a significant decrease in *Candida albicans* adhesion with the addition of NDs in comparison with the control group. The first research hypothesis was partly rejected because the surface roughness of the NDs-PMMA groups was significantly reduced compared to the control group, while the contact angle values showed no significant change. The second research hypothesis was rejected because NDs-PMMA at different concentrations of NDs presented antifungal activity against *Candida albicans*.

Surface roughness has been shown to be directly related to the number of microorganisms deposited on the denture surface.^31^ A denture base with a higher Ra provides a greater surface area for colonization.^31^ The present study indicated a significant decrease in the *Candida albicans* count in the NDs groups compared with the control group, which had the highest Ra value. According to the surface roughness test and SEM analysis, the addition of NDs to PMMA improved the surface structure of the specimens, which could contribute to the prevention of *Candida albicans* adhesion. Compared to the control group, SEM analysis showed that the addition of NDs changed the surface profile of the tested specimens by filling the pores. After complete saturation, NDs formed clusters on the surface of specimens. The reduced surface roughness may have resulted from the particles’ small size, which reduced the inter-particle distance, resulting in close contacts between the nanoparticles at lower concentrations.^32^ Moreover, NDs have multiple reactive groups (NH_2_,OH), which improve their interfacial bond with PMMA.^24^ However, an increase in Ra was observed with a high concentration of NDs (1.5%), but was still lower than in the control group and the clinically acceptable value (0.2 μm)^16^ This increase may have resulted from spaces created on the surface of specimens due to the separation of loosely attached clusters of NDs particles after finishing and polishing.

These results are in line with those of a previous study that reported lower porosity of barium titanate nanocomposites.^32^ Lainović et al.^33^ found that nanofiller-containing composites allow for a more uniform surface topography after polishing. The reduced Ra of PMMA incorporating NDs may have been a consequence of the improved polishability due to the presence of small nanofillers merging within the denture base resin, providing a smooth polished surface.^33^ These findings were consistent with findings of previous studies which deduced that the homogeneous distribution of nanofiller significantly decreases surface damage to the resulting nanocomposites.^34^^,^^35^ On the other hand, a preceding study reported a significant increase in the surface roughness of PMMA modified with a nanofiller, but still remained below the clinically accepted Ra.^36^

The addition of NDs to PMMA showed no significant change in the contact angles of the specimens containing NDs in comparison to the control group. This result diverges from several studies that reported changes in the PMMA contact angle with the addition of nanofillers. Studies show that the addition of nanographene oxide to PMMA and zinc oxide polymethyl methacrylate nanocomposites have increased the hydrophilicity of the resin with reduced potential for microbial adhesion.^37^^,^^38^ Similarly, reduced hydrophobicity of denture base resin was reported with silver nanoparticles, but microbial adhesion was unaffected.^39^ Hashem, et al.^40^ (2017) suggested that the addition of titanium oxide nanoparticles to PMMA improves its wettability. However, Kim, et al.^41^ (2019) found an increase in the water contact angle with increasing carbon nanotube content in PMMA. Their study demonstrated that an increase in the contact angle increases the material's surface hydrophobicity, enhancing*Candida albicans* adhesion.^42^ Several studies tested the effect of the contact angle on *Candida albicans* adhesion and reported varied findings.^29^^,^^43^ Some authors found that the contact angle significantly affected *Candida albicans* adhesion,^43^^,^^44^ while others discovered an association between them.^29^^,^^45^ Murat, et al.^29^ (2019) and Serrano-Granger, et al.^45^ (2005) identified no association between contact angle and *Candida albicans* adhesion, but found a significant correlation with surface roughness, which agree with the results of the present study.

Neubauer and CFU tests showed a significant decrease in the *Candida albicans* count in the NDs groups compared with the control group. The lowest *Candida albicans* count in both tests occurred in the 1% NDs group. The effect of NDs against *Candida albicans* adhesion had no previous investigation, but NDs may act against fungi in a similar manner as against bacteria. Several studies have reported the antibacterial activity of NDs.^25^^–^^27^^,^^46^ The antibacterial effect of NDs was attributed to the activity of ND particles and their ability to surround the bacterial cells, thereby blocking essential cell functions.^23^ Researchers also suggested that the antibacterial effect is linked to the presence of partially oxidized and negatively charged surface functions, especially acid anhydride groups, which enhance the NDs’ antibacterial activities.^26^ Moreover, NDs have been described among anti-adhesive nanoparticles that inhibit biofilm formation.^27^^,^^47^

Generally, adding nanoparticles improves overall performance of PMMA/filler nanocomposites.^19^^,^^30^^,^^32^ The addition of NDs has demonstrated to improve mechanical properties of PMMA.^28^ Clinically, NDs could be added to PMMA due to their potential to reduce *Candida albicans* adhesion. Unfortunately, a noticeable color change was observed in the tested specimens, particularly at higher concentrations of NDs. To overcome this problem, the denture could be made in two layers by adding NDs to form the intaglio surface of the maxillary denture or to unaesthetic areas. Furthermore, NDs could be added to relining and tissue conditioning materials to benefit from its effect against *Candida albicans* adhesion. Durability of the effect of the PMMA/ND composite would improve denture hygiene, especially among patients with physical disabilities. Further, long-term investigations are needed to test the durability of the effect of NDs, the mechanism of the antifungal effect when using different concentrations of NDs, and different acrylic resin brands.

Strong points of this analysis include using two methods to assess the *Candida albicans* count and using SEM to examine surface characteristics of PMMA denture base material. Limitations of this study include testing the specimens in conditions that diverge with the oral cavity environment in terms of changes in pH, temperature, and the presence of natural saliva and other microorganisms.

## Conclusion

The study found that the addition of NDs to PMMA acrylic denture base material significantly decreased *Candida albicans* adhesion and surface roughness, with the lowest values observed at 1% NDs and 0.5% NDs, respectively, while no significant effect was observed on the contact angle. The addition of NDs to denture base resin could be a possible contributor to DS prevention.
